# Clinical Relevance of Drug Interactions in People Living with Human Immunodeficiency Virus on Antiretroviral Therapy—Update 2022: Systematic Review

**DOI:** 10.3390/pharmaceutics15102488

**Published:** 2023-10-18

**Authors:** Pedro Amariles, Mónica Rivera-Cadavid, Mauricio Ceballos

**Affiliations:** 1 Research Group on Pharmaceutical Promotion and Prevention, University of Antioquia, UdeA, AA 1226, Medellin 050010, Colombia; mpatricia.rivera@udea.edu.co (M.R.-C.); javier.ceballos@udea.edu.co (M.C.); 2 Research Group on Pharmaceutical Care, University of Granada, 18071 Granada, Spain; 3 Research Group on Pharmacy Regency Technology, University of Antioquia, Medellin 050010, Colombia

**Keywords:** drug interactions, antiretroviral agents, HIV/AIDS

## Abstract

Background: The clinical outcomes of antiretroviral drugs may be modified through drug interactions; thus, it is important to update the drug interactions in people living with HIV (PLHIV). Aim: To update clinically relevant drug interactions in PLHIV on antiretroviral therapy with novel drug interactions published from 2017 to 2022. Methods: A systematic review in Medline/PubMed database from July 2017 to December 2022 using the Mesh terms antiretroviral agents and drug interactions or herb–drug interactions or food–drug interactions. Publications with drug interactions in humans, in English or Spanish, and with full-text access were retrieved. The clinical relevance of drug interactions was grouped into five levels according to the gravity and probability of occurrence. Results: A total of 366 articles were identified, with 219 (including 87 citation lists) were included, which allowed for the identification of 471 drug interaction pairs; among them, 291 were systematically reported for the first time. In total 42 (14.4%) and 137 (47.1%) were level one and two, respectively, and 233 (80.1%) pairs were explained with the pharmacokinetic mechanism. Among these 291 pairs, protease inhibitors (PIs) and ritonavir/cobicistat-boosted PIs, as well as integrase strand transfer inhibitors (InSTIs), with 70 (24.1%) and 65 (22.3%) drug interaction pairs of levels one and two, respectively, were more frequent. Conclusions: In PLHIV on antiretroviral therapy, we identify 291 drug interaction pairs systematically reported for the first time, with 179 (61.5%) being assessed as clinically relevant (levels one and two). The pharmacokinetic mechanism was the most frequently identified. PIs, ritonavir/cobicistat-boosted PIs, and InSTIs were the antiretroviral groups with the highest number of clinically relevant drug interaction pairs (levels one and two).

## 1. Introduction

Human immunodeficiency virus (HIV) is one of the main public health problems. According to the World Health Organization (WHO), globally, 39.0 million (33.1–45.7 million) people were living with HIV at the end of 2022; additionally, during 2022, 630,000 (480,000–880,000) people died from HIV-related causes and 1.3 million (1.0–1.7 million) persons acquired HIV [1]. In recent years, remarkable advances have been achieved in the treatment of HIV; thus, currently, most people living with HIV (PLHIV) have a life expectancy similar to persons without HIV. According to the latest updated guidelines, it is recommended to start antiretroviral (ARV) therapy as soon as possible after HIV diagnosis, ideally within 7 days. Additionally, if they have an opportunistic infection, ARV therapy should be started shortly after the initiation of the treatment for the infection, being recommended within 2 weeks [2].

Currently, in PLHIV, initial ARV therapy generally consists of two nucleoside/nucleotide reverse transcriptase inhibitors (NRTIs) combined with a third active ARV drug, which may be an integrase strand transfer inhibitor (InSTI), a non-nucleoside reverse transcriptase inhibitor (NNRTI), or a protease inhibitor (PI) boosted with cobicistat (COBI) or ritonavir (RTV). InSTIs such as bictegravir (BIC) or dolutegravir (DTG) are the preferred third ARV drug, mainly due to being associated with a lower risk for drug resistance and for drug–drug interactions [2]. Additionally, the two-drug regimen, DTG plus lamivudine (3TC), may be recommended for the initial option for patients with an initial HIV viral load of <500,000 copies/mL; for patients who have achieved viral suppression, a long-acting injectable regimen of bimonthly injections of long-acting cabotegravir (CAB) and rilpivirine (RPV) may be used. Additionally, advances in ARV therapy have led to the availability of well-tolerated single-tablet regimens that are associated with a lower risk of drug interactions, as well as options for pre-exposure prophylaxis, including daily oral medications, such as tenofovir (TDF)/emtricitabine (FTC), or bimonthly injectable CAB [2].

A drug interaction is an undesirable modification that is quantifiable in the magnitude or duration of effects related to the simultaneous or previous administration of other drugs, phytotherapeutics, foods, or due to pathophysiological (special) conditions of the patient [3]. The identification, prevention, and resolution of clinically relevant drug interactions are a critical aspect of achieving pharmacotherapy goals. Among other methods for evaluating the clinical relevance of interactions, a proposal based on the gravity of the effect on the patient’s health (grave, moderate, and minor) and the probability of occurrence (defined, probable, and possible, according to the type of study supporting the drug interaction) has been considered as appropriate. This proposed classification generates four levels of clinical relevance: level one (very high risk), level two (high risk), level three (medium risk), and level four (low risk) [3,4]. In addition, a new level of clinical relevance (level five: lowest risk) has been proposed, which is characterized by the absence of an effect on the patient’s health (lack of gravity) documented in meta-analyses, systematic reviews, or clinical trials (defined probability), and, therefore, with evidence of the absence of clinically relevant drug interactions [4].

Regarding clinically relevant drug interactions in persons with HIV, from 1995 to 2017, we identified four previously published reviews, which focused on identifying drug interactions between ARV drugs, phytotherapeutics, and foods [5,6,7,8]. The most recent review updated the reported ARV interactions up to June 2017 [8]. However, due to the commercialization of new ARVs, updates to guidelines and expert recommendations and, mainly, both the identification and reporting of new clinically relevant drug interactions or the generation of new knowledge about drug interactions systematically reported previously, this information should be periodically updated. In addition, in 2017, a free software to facilitate the identification and assessment of the clinical relevance of ARV drug interactions (SIMARV^®^) was developed [9]; then, a free mobile version (InterApp-ARV) was developed and is available for mobile phones and tablets [10]. The development of both SIMARV^®^ and InterApp ARV, used as a graphic reference the free software developed by the University of Liverpool (https://www.hiv-druginteractions.org/ accessed on 25 July 2023), is considered as the most used online source of DDI in HIV [11]. In this context, this systematic review aimed to update clinically relevant drug interactions in PLHIV on antiretroviral therapy, with novel drug interaction pairs between ARVs and other medications, phytotherapeutics, or foods published from 2017 to 2022.

## 2. Materials and Methods

Similar to previously published reviews, a systematic review was conducted in the Medline/PubMed database from 1 July 2017 to 31 December 2022 using the Mesh terms antiretroviral agents AND drug interactions OR herb–drug interactions OR food–drug interactions. Articles published in English or Spanish and with full-text access were identified.

Inclusion criteria: We included all articles containing clinically relevant information on drug interactions in humans using antiretroviral agents for the treatment of persons living with HIV/AIDS. Additionally, other studies were identified from the reference list of retrieved articles.

Exclusion criteria: We excluded the following types of articles: (a) preclinical or in vitro studies; (b) with theoretical concepts regarding drug interactions; (c) without specific ARV drug interactions; (d) not related to HIV; and (e) without full-text availability.

To ensure a systematic approach, three researchers reviewed the studies identified according to the preferred reporting items for systematic reviews and meta-analysis (PRISMA) flow chart via a predetermined eligibility criteria [12]. The titles and abstracts of all identified articles were screened for eligibility by the three authors, and any discrepancies were resolved by consensus. Subsequently, to allow for the synthesis and analysis of the results, the data were collected in a table with the following information: (a) article title, ARV assessed, (b) drug-related interaction, (c) clinical relevance level (according to the combination of the gravity and probability of occurrence), (d) pharmacodynamic or pharmacokinetic mechanism, (e) comment and recommendation, and (f) reference. The information registered was proofread by the three authors.

The drug interaction pairs of identified ARV agent–drug interactions were classified into five levels according to the gravity (effect on patient’s health) and probability of occurrence (type of study that supports the interaction), following the combination of options, as shown in Table 1 [3,4].

The probability was determined and classified according to the kind of study that supported the interaction found for each pair of drug interactions [3]:

- Possible: The drug interaction pair was documented with results from less than three case reports or by expert consensus.
- Probable: The drug interaction pair was documented with results from at least one observational study (cohort or case–control study) or at least three case reports.
- Defined: The drug interaction pair was documented with results from at least one meta-analysis, systematic review, or randomized or nonrandomized clinical trial.

In the cases of the reviews, including systematic reviews or meta-analyses, the reference list was reviewed and the drug interaction had to be support with a clinical study. In addition, if the study (case reports, observational study, clinical trial, systematic review, or meta-analysis) was identified for the first time, it was included. Therefore, in the current update, the drug interaction pair probability, due to bringing together all the references that supported it, could be (a) systematically identified for the first time (the references identified for the first time generated the probability), (b) increased (the references identified for the first time modified the probability), or conserved (the references systematically identified for the first time reinforced it but did not modify it).

The gravity attributed to the drug interaction was determined and classified according to the effect on the patient’s health [3,4]:

- Lack of gravity: There was evidence that the drug interaction did not cause harm to the patient.
- Minor: The drug interaction did not cause or caused minimum harm to the patient (including those that did not require an additional drug treatment nor generated qualitative or quantitative pharmacotherapy changes, neither increasing the patient’s hospitalization), but generated the need for monitoring the patient’s health.
- Moderate: The drug interaction generated the need for a closer monitoring of the patient’s health (including those that required an additional drug treatment, generated qualitative or quantitative pharmacotherapy changes, or increased the patient’s hospitalization.
- Grave: The drug interaction could cause harm or injury to the patient (including those that could be life threatening, result in persistent or significant disability or hospitalization, or cause birth defects).

## 3. Results

**Figure 1:**
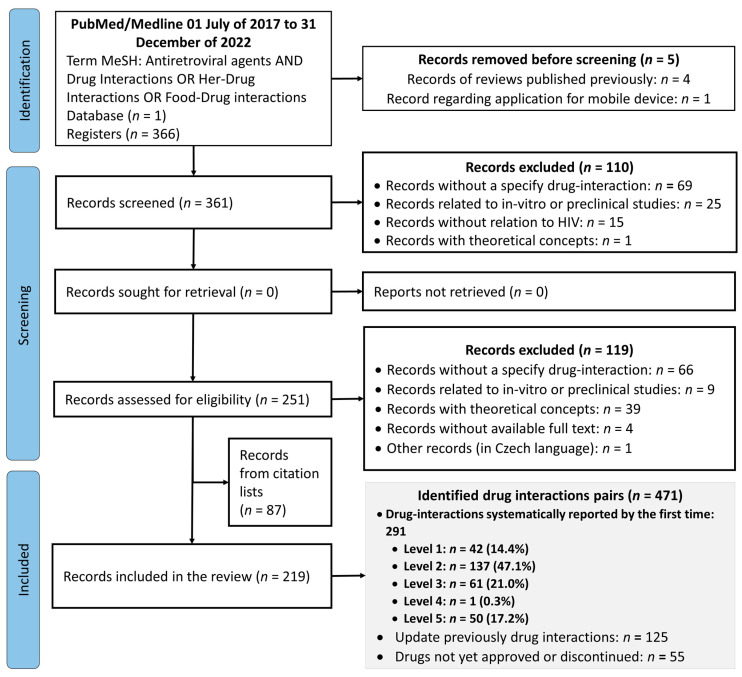
Preferred reporting items for systematic review and meta-analysis (PRISMA) [12] flow diagram for the systematic review of the clinical relevance of drug interactions in people living with human immunodeficiency virus.

From the search in the PubMed/Medline database, 366 records were retrieved; among them, 5 were removed before screening. Then, 110 records were excluded due to the screened title and abstract. Thus, 251 articles were assessed for eligibility; among them, 119 were excluded and, consequently, a total of 132 articles were included in the review. In addition, from the citation list, 87 articles were included; thus, 219 articles were used for this review (Figure 1). However, in the current article, only drug interactions assessed as levels one, two, and five were presented, which were supported by 194 [13,14,15,16,17,18,19,20,21,22,23,24,25,26,27,28,29,30,31,32,33,34,35,36,37,38,39,40,41,42,43,44,45,46,47,48,49,50,51,52,53,54,55,56,57,58,59,60,61,62,63,64,65,66,67,68,69,70,71,72,73,74,75,76,77,78,79,80,81,82,83,84,85,86,87,88,89,90,91,92,93,94,95,96,97,98,99,100,101,102,103,104,105,106,107,108,109,110,111,112,113,114,115,116,117,118,119,120,121,122,123,124,125,126,127,128,129,130,131,132,133,134,135,136,137,138,139,140,141,142,143,144,145,146,147,148,149,150,151,152,153,154,155,156,157,158,159,160,161,162,163,164,165,166,167,168,169,170,171,172,173,174,175,176,177,178,179,180,181,182,183,184,185,186,187,188,189,190,191,192,193,194,195,196,197,198,199,200,201,202,203,204,205,206] of those 219 articles.

A total of 471 drug interaction pairs between antiretroviral agents and other drugs were identified; of them, 291 were interactions systematically reported for the first time, 125 were updates to drug interactions reported previously, and 55 were related to drugs not yet approved or to discontinued drugs in clinal practice (Figure 1). The clinical relevance levels (based on gravity and probability) and the mechanism for the 291 pairs of drug interactions systematically reported for the first time are shown in Table 2.

Among the 291 interactions systematically reported for the first time, 179 (61.5%) were assessed as having a higher risk of causing adverse drug outcomes related to the ineffectiveness or unsafe use of the pharmacotherapy, of which 42 (14.4%) and 137 (47.1%) were assessed as level one and level two, respectively (Table 2). Table 3 and Table 4 provide detailed information for the drug interaction pairs assessed as levels one and two. Among the ARV pharmacologic groups, the protease inhibitors (PIs) and RTV/COBI-boosted PIs, the integrase strand transfer inhibitors (InSTIs), and the non-nucleoside reverse transcriptase inhibitors (NNRTIs) with 70 (24.1%), 65 (22.3%), and 37 (15.9%) drug interaction pairs of levels one and two, respectively, were the more frequent (Table 3 and Table 4). In addition, for 233 (80.1%) of the 291 drug interaction pairs, the pharmacokinetic mechanism was the most frequent, including bioavailability modifications based on P-gp or presystemic enzyme alterations (Table 2).

Globally, among the 291 interactions systematically reported for the first time, 50 (17.2%) were assessed as being level five; thus, with evidence of the absence of clinically relevant drug interactions, 20 (6.1%) for InSTIs, 14 (4.8%) for NNRTIs, and 9 (3.1%) for PIs and RTV/COBI-boosted PIs were the more frequent (Table 5). In addition, information regarding 55 drug interaction pairs was related to drugs not yet approved or to discontinued drugs or with minimum use in clinal practice, with 43 corresponding to PIs (indinavir, nelfinavir, saquinavir, tipranavir, and fosamprenavir), 11 corresponding to NRTIs (didanosine and stavudine), and 1 to a sulfonylurea (chlorpropamide).

Among 179 drug interactions of levels one and two, cation-containing antacids/supplements and iron or zinc products, antiulcer (proton pump inhibitors and anti-H_2_), with 29, (16.2%) and oral anticoagulants (warfarin and direct-anticoagulants), with 23 (12,8%), were the non-ARV pharmacologic groups with more drug interaction pairs. Additionally, there were six (3.4%) clinically relevant drug interactions (level two) associated with drug or substance misuse, such as amphetamines or ketamine, mainly due to the concomitant use of boosted PIs and elvitegravir/cobicistat (EVG/COBI). Additionally, six (3.4%) drug interaction pairs with benzodiazepines were identified (Table 3 and Table 4).

## 4. Discussion

Although current ARV therapy is simplified, safe, and effective, the combination of three ARV drugs or two with some recent regimens [2] increases the risk of clinically relevant drug interactions, mainly in patients with one or more long-term disease in addition to HIV. Currently, some systematic reviews, meta-analyses, and randomized clinical trials provide sufficient evidence that some drug interactions may cause negative health outcomes in PLHIV. Overall, clinically relevant drug interactions occur in 20–30% of PLHIVs [207], including recently authorized ARV drugs, mainly explained through pharmacokinetics changes linked to the inhibition or induction of different enzymes and metabolic transporters [3,5,6,7,8,208].

The current review identified 219 articles with information that allowed us to assess and classify the clinical relevance of 471 drug interaction pairs in PLHIVs on antiretroviral therapy. Among these 471 drug interaction pairs, 291 were systematically reported for the first time, which shows the need for conducting a periodic update on this topic through comprehensive reviews [5,6,7,8].

In the current update, we identified 291 drug interaction pairs systematically reported for the first time; among them, 179 (61.1%) were assessed as the most clinically relevant (levels one or two). These figures were lower than reported in the update for 2015–2017 [8], in which 534 drug interaction pairs were systematically reported for the first time and 308 (64.2%) were assessed as levels one or two. This reduction may be due to the current guidelines [2] recommending, as first therapeutic options, regimes with more recent ARV drugs, such as BIC (authorized in 2018) or CAB (authorized in 2021), which have less probability of pharmacokinetic interactions. In this sense, among 50 interaction pairs assessed as level five, 20 (40.0%) were related to InSTIs (Table 5). Overall, second-generation InSTIs (CAB, BIC, and DTG), compared to PIs, have less probability to inhibit the metabolism of CYP450 isoenzymes and, therefore, a lesser risk of clinically relevant drug interactions [2,209]. Similarly, for NNRTIs, 14 (28.0%) drug interaction pairs were assessed as level five, which may be explained due to more recent drugs (second generation), for instance, doravirine (DOR), etravirine (ETR), and rilpivirine (RPV) may have less probability of pharmacokinetic interactions in comparison with nevirapine (NVP) and efavirenz (EFV) [5,6,7,8].

The pharmacokinetic mechanism was the most frequent to explain the drug interaction; thus, it was the mechanism to 233 (80.1%) of the 291 pairs systematically reported for the first time. The pharmacokinetics of ARV involve the isoenzymes of the cytochrome P450 enzyme (CYP) family, such as CYP3A4, CYP2B6, and CYP2C9, enzyme of the glucuronidation pathway, such as UGT1A1, as well as uptake transporters, such as OCT2, MATE1, OATP1B1, and export proteins, such as glycoprotein P (P-gp), which increase the probability of pharmacokinetic drug interactions [210]. Some ARV drugs, especially PIs and NNRTIs, are considered strong inhibitors or inducers of various isoenzymes of the CYP450 family, as well as carrier proteins, which increase the risk of significant clinical drug interactions, thereby increasing the risk of not achieving the therapeutic goals in PLHIVs [205].

In the current review, the ARV groups with more clinically relevant drug interaction pairs were the PIs and RTV/COBI-boosted PIs, the InSTIs, and the NNRTIs, with 70 (24.1%), 65 (22.3%), and 37(15.9%) drug interaction pairs of levels one and two, respectively, for PIs and RTV/COBI-boosted PIs and NNRTIs, likely due to their metabolism through the CYP450 family [210], and for InSTIs, mainly related to EVG/COBI, likely due to both metabolism through the CYP3A4 and the inhibition of CYP3A4 activity through COBI. Similarly, a retrospective study found that the most frequently involved ARVs were RTV/COBI-enhanced PIs (49.3%), followed by NNRTIs (38.3%) [211]. However, these results were slightly different from the findings in the 2015–2017 update, in which PIs were the predominant group with a percentage of 29.2% [8].

Drug substance misuse is an important consideration in PLHIV on ARV therapy, requiring an integrated approach based on evidence [2]. Therefore, there are clinically relevant drug interactions associated with drug substance misuse, such as ketamine, amphetamine, and substitutes (methamphetamine, methylenedioxymethamphetamine—MDMA; ‘ecstasy’) with RTV-boosted PIs [14,75,76,77,78,79,80] and EVG/COBI [14,15,75,76,80,142,143], increasing the risk of toxicity, including a possible fatal serotonergic reaction. Furthermore, clinically relevant interactions were identified between psychotropic drugs, particularly benzodiazepines, which have the potential to cause dependence in patients. In combination with boosted PIs and EVG/COBI, the probability of respiratory depression, sedation, and muscle weakness was increased [13,14,15,93]. It is important to denote that, currently, there is increasing awareness about the probability occurrence of clinical relevance of cannabis–drug interactions [212], particularly with efavirenz (EFV) and COBI or RTV-boosted regimes, which may increase plasma levels of cannabis, prolonging its clinical effects and increasing toxicity [33].

The method for assessing the clinical relevance of drug interactions used in this review was similar to that used in previous reviews [5,6,7,8]; therefore, these results were useful in updating and synthesizing the previous identified information regarding ARV drug interactions. Thereby, the 291 drug interaction pairs systematically reported for the first time should be used to update the mobile application for analyzing the clinical relevance of ARV drug interactions (InterApp ARV) [10], which is an evolution of the SIMARV^®^ software [9]. The application is freely accessible and can be downloaded for Android devices from the Play Store (https://play.google.com/store/apps/details?id=co.com.pypudea.interapparv accessed on 25 July 2023).

Neither the current nor the previous reviews included the specific search for pharmacogenetic interactions [5,6,7,8], mainly, gene–antiretroviral drug interactions. However, this issue emerges as a key explanation for clinically relevant drug interactions. For instance, patient genetics explain the extent of the inductor effect of efavirenz or nevirapine on etonogestrel pharmacokinetics, and show that drug interactions with NNRTIs are influenced by host genetics. Thus, the combination of efavirenz plus etonogestrel/ethinylestradiol (vaginal ring) results in an unfavorable drug–drug interaction regardless of patient genetics (Table 3), but it is most notorious in women with variant alleles for CYP2B6 single-nucleotide polymorphisms (slow metabolizer genotype) [60,61]. As a consequence, this issue should be included in future systematic reviews.

The results of this review may have some limitations; therefore, the results should be interpreted and used with caution. In this context, the main limitation was the search restriction to a single database, since the search was performed only in the PubMed/MEDLINE database, which may not have identified other clinically relevant interactions. However, this situation could be minimized with the inclusion of publications identified as relevant in the reference list of the articles included. In addition, the method for assessing and classifying the level of relevance could be considered a subjective scale of “clinical significance”, which was not subject to a validation process. However, the method was proposed in 2007 [3] and updated in 2021 [4], and among several reviews of clinically relevant drug interactions, it was used for four previous reviews regarding antiretroviral drug interactions [5,6,7,8] for other pharmacologic groups, for instance, cannabis [212], or for hypolipidemic agents [213] and for specific drug–drug interactions, for instance, for nonsteroidal anti-inflammatory drugs and antihypertensives [214]. Additionally, it was used for assessing drug interactions in different settings, for instance, in intensive care [215].

## 5. Conclusions

From 2017 to 2022, in the PubMed/Medline database, we identified 219 records, including 87 from citation lists, related to 291 drug interaction pairs in PLHIVs in patients living with HIV on antiretroviral therapy. Thus, the clinical relevance of 471 drug interaction pairs was assessed; of them, 291 were pairs systematically reported for the first time, a figure that was lower than the one reported in the update for 2015–2017. Among the 291 drug interaction pairs systematically reported for the first time, 179 (61.5%) were assessed as level one (42) or level two (137), thus, with a high risk of causing adverse drug outcomes linked to ineffectiveness or unsafety of the pharmacotherapy. Pharmacokinetics is the mechanism most frequently identified to explain drug interactions. In addition, PIs and RTV/COBI-boosted PIs and InSTIs were the ARV drugs with a greater number of clinically relevant interactions. Cation-containing antacids/supplements and iron or zinc products, antiulcer (proton pump inhibitors and anti-H2), and oral anticoagulants were drug groups most frequently with drug interactions with ARV, accounting for 16.2% and 12.8% of cases, respectively. In PLHIVs, clinically relevant drug interactions were associated with drug substance misuse, mainly amphetamines and psychotropic drugs, particularly benzodiazepines.

## Figures and Tables

**Table 1:** Levels of the clinical relevance of drug interactions according to the combination of gravity and probability of occurrence [3,4].

Probability			
**Gravity**	Defined	Probable	Possible
**Grave**	1 (very high risk)	1 (very high risk)	2 (high risk)
**Moderate**	2 (high risk)	2 (high risk)	3 (medium risk)
**Minor**	3 (medium risk)	3 (medium risk)	4 (low risk)
**Lack of gravity**	5 (lowest risk)	N/A	N/A

N/A: Not applicable.

**Table 2:** Summary of 291 antiretroviral–drug interactions systematically reported for the first time.

Total of Drug Interaction Pairs, *n* (%)					291 (100%)
**Pharmacokinetic mechanism**					**233 (80.1%)**
Enzyme inhibition					101 (43.3%)
Enzyme induction					71 (30.5%)
Bioavailability modifications based on pH alteration or chelation					19 (8.2%)
Bioavailability modifications based on P-gp or presystemic enzyme alterations					39 (16.7%)
Protein displacement					1 (0.4)
Bidirectional enzyme inhibition/induction					2 (0.9%)
**Pharmacodynamic mechanism**					**5 (1.7%)**
Synergism (toxicity)					5 (100%)
**Pharmacokinetic/pharmacodynamic mechanism**					**3 (1.0%)**
Enzyme inhibition/synergism					2 (66.7%)
Enzyme induction/synergism					1 (33.3%)
**Evidence of absence of clinically relevant drug interactions**					**50 (17.2%)**
**Level of the clinical relevance of drug interaction**					
**Level 1**	**Level 2**	**Level 3**	**Level 4**	**Level 5**	**Total, *n* (%)**
42 (14.4%)	137 (47.1%)	61 (21.0%)	1 (0.3%)	50 (17.2%)	291 (100.0%)

**Table 3:** Drug interaction pairs with protease inhibitors and non-nucleoside reverse transcriptase inhibitors, levels 1 and 2, systematically reported for the first time.

Pharmacological Group or Drugs Affected	Antiretroviral Agent	Gravity/Probability	Comments/Suggestions
Anesthetic/anxiolytic/benzodiazepine			
Midazolam (oral) [13,14,15]	ATV/RTV	(Grave/Possible): Level 2	Boosted PIs may increase both midazolam plasma levels and the probability of respiratory depression, sedation, and muscle weakness. Overall, the use of oral midazolam with PIs is not recommended. Additionally, parenteral midazolam has been associated with the risk of severe prolonged sedation in a cohort of hospitalized HIV patients. Thus, this combination should be closely monitored or alternative sedatives for procedural sedation considered (lorazepam or propofol).
Midazolam (intravenous) [13,14,15,16]		(Grave/Probable): Level 1	
Triazolam [13,14,15]	ATV/RTV	(Grave/Possible): Level 2	Boosted PIs may increase both triazolam plasma levels and the probability of respiratory depression, sedation, and muscle weakness. Overall, the use of oral midazolam with PIs is not recommended. Additionally, consider using other safer alternatives, such as oxazepam, lorazepam, or temazepam.
**Antiasthmatic/glucocorticoid**			
Budesonide [13,14,15,16]	ATV	(Grave/Possible): Level 2	ATV may increase the budesonide plasma levels, enhancing the side effects produced by steroids. Monitoring budesonide safety parameters and a dose adjustment may be necessary.
Fluticasone [17]	LPV/RTV	Grave/Possible): Level 2	LPV/RTV may increase the fluticasone plasma levels (due to CIP3A4 inhibition), which can cause isolated myopathy (unusual manifestation of inhaled fluticasone). This combination should be avoided. If it is needing, beclomethasone and budesonide are considered as much safer alternatives. Additionally, similar to the COBI-based regimen, consider switching RTV-boosted regimens to BIC-, DTG-, RAL-, or RPV-based regimens.
**Anticonvulsant**			
Carbamazepine–oxcarbazepine [14,15,16,18]	ATV	(Moderate/Probable): Level 2	Carbamazepine or oxcarbazepine may cause a significantly lower trough concentration of ATV by 65% (190 ± 91 vs. 546 ± 380 ng/mL), and may be associated with the induction of CYP3A4 and UGT1A1 (metabolizing enzymes). Thus, these combinations should be avoided, owing to the potential risk of virological failure.
**Antimalarial**			
Atovaquone [19,20,21,22]	EFV, ETR	(Moderate/Defined): Level 2	EFV or ETR may decrease the AUC and C_max_ of atovaquone by inducing glucuronidation. Monitoring atovaquone effectiveness parameters and a dose adjustment may be necessary.
Proguanil [20,23,24]	EFV, ETR	(Moderate/Defined): Level 2	EFV or ETR may decrease proguanil AUC and C_max_. Monitoring proguanil effectiveness parameters and a dose adjustment may be necessary.
Atovaquone/proguanil [15,19,20,21,22,23,24,25]	EFV	(Moderate/Defined): Level 2	EFV-based antiretroviral therapy decreases AUC atovaquone/proguanil by 75% and proguanil AUC by 44%, leading to possible atovaquone/proguanil prophylaxis failures in HIV-infected patients. Thus, these combinations should be used with caution or avoided. Additionally, an increase in the dose of atovaquone/proguanil or other alternative drug for malaria prophylaxis may be considered.
Artemether/lumefantrine [26]	ATV/RTV	(Moderate/Defined): Level 2	ATV/RTV may significantly increase QTc interval from 0.4079 ± 0.008 to 0.4215 ± 0.007 s, leading to artemether/lumefantrine, potentially being cardiotoxic.
Artesunate/amodiaquine [27]	NVP	(Moderate/Probable): Level 2	Coadministration of nevirapine and artesunate/amodiaquine may be associated with hepatoxicity and may be associated with synergic hepatotoxicity effect (pharmacodynamic mechanism). Thus, the use of combination artesunate/amodiaquine and NVP should be avoided or be used with a careful monitoring of liver function.
Mefloquine [28]	NVP	(Moderate/Probable): Level 2	Mefloquine may cause a decrease in exposure to NVP (concentrations are significantly lower in both maternal plasma and cord plasma, which may increase the risk of mother-to-child transmission among HIV-positive women receiving this combination). Thus, this combination should be avoided.
Artemether/lumefantrine [29,30,31,32,33]	EFV	(Moderate/Defined): Level 2	EFV-based antiretroviral therapy may decrease artemether/lumefantrine exposure (47% for lumefantrine) in pregnant and not pregnant women with malaria, which is more pronounced in CYP2B6 slow metabolizers (CYP2B6*6/*6 genotype, linked to two- or three-fold higher EFV plasma levels). Thus, mainly pregnant women or persons with the CYP2B6*6/*6 genotype, may receive subtherapeutic doses due to a higher EFV effect, and, thereby, a dose adjustment and monitoring of artemether/lumefantrine effectiveness may be necessary. Additionally, the EFV effect may be attenuated by using LPV/TRV in conjunction with the NVP-based ART regimen.
**Antimicrobial/antituberculosis**			
Moxifloxacin [34]	EFV	(Moderate/Defined): Level 2	EFV may increase moxifloxacin oral clearance by 42%, resulting in a 30% reduction in moxifloxacin AUC, and increasing the risk of resistance and antimicrobial failure. When coadministered with efavirenz, it is necessary to increase the moxifloxacin doses to maintain moxifloxacin levels within the stablished therapeutic range. Additionally, levofloxacin is suggested instead of moxifloxacin when used concomitantly with EFV.
Rifabutin [13,15,35,36,37,38]	DOR	(Moderate/Defined): Level 2	Rifabutin may decrease DOR plasma levels through the induction of CYP3A4. Doravirine 100 mg twice daily may be necessary and also to monitor DOR effectiveness parameters.
Rifabutin [13,35,39]	EFV, ETR	(Moderate/Defined): Level 2	EFV or ETR may induce CYP3A4 and increase rifabutin metabolism, causing decrease in their plasma levels and response. Thus, daily rifabutin may be increase by 50% (300 mg once daily).
Rifabutin [13,15,35,40,41,42]	RPV	(Moderate/Defined): Level 2	Rifabutin may decrease RPV AUC and C_max_ by 46% and 35%, respectively, increasing the risk of virological failure and the development of resistance; therefore, this combination is not recommended, mainly in parenteral RPV. Additionally, rifabutin 300 mg daily and a double dose of oral RPV (50 mg) may be used.
Rifampicin [13,34,38,43]	EFV	(Moderate/Defined): Level 2	Rifampicin may induce CYP3A4 and decrease EFV exposure and virologic response; thus, although the coadministration of rifampicin and EFV could be safe and effective, the dose of EFV may be increased from 400 to 600 mg once daily (800 mg once daily if patient weigh is >60 kg); it is important to monitor EFV serum levels and, if needed, to adjusted doses to obtain concentrations within the reference range (1–4 mg/L). In addition, if it is possible, the EFV dose may be defined according to the CYP2B6 metabolizer genotype of the patient (slow or extensive). Additionally, EFV may induce CYP3A4 and decrease rifampicin exposure and microbiologic response; thus, the daily dose of rifampicin may be increased by 50% in the presence of EFV (intervention that has a minor effect on EFV concentrations and is well tolerated).
Rifampicin [13,15,21,35,44]	DRV/RTV	(Grave/Defined): Level 1	Rifampicin may significantly decrease DRV/RTV plasma levels by more than 98% (similar to other PIs), increasing the risk of the development of resistance and virological failure. The DRV/RTV (similar to other PIs/RTV) dose increase does not warrant satisfactory DRV exposures and may cause severe alanine transaminase elevations and excessive risk of hepatotoxicity. Alternatively, consider a rifabutin 150 mg daily dose.
Rifampicin [13,15,35,36,37,42,45]	DOR	(Grave/Defined): Level 1	Long-term coadministration of rifampicin may induce CYP3A4, causing a decrease in DOR plasma levels by 88%, and may reduce its efficacy. Administration of rifampicin with DOR is not recommended.
Rifampicin [13,15,35,40,41,43,46]	RPV	(Grave/Defined): Level 1	Rifampicin may decrease RPV AUC and C_max_ by 80% and 69%, respectively, increasing the risk of virological failure and the development of resistance; therefore, this combination is contraindicated. As an alternative, daily rifabutin at 300 mg and a double dose of RPV are recommended.
Rifampicin [15,47]	ETR/TAF	(Moderate/Defined): Level 2	Rifampicin may decrease TAF exposure when ETR/TAF is coadministered; however, intracellular tenofovir diphosphate (DP) concentrations are 4.21 higher when TAF is coadministered with rifampin than when TDF is administered alone, although clinical outcomes have not been studied. Thus, this combination is not recommended; however, if it is coadministered, monitor virologic response.
**Antineoplastic**			
Venetoclax [48]	RTV	(Moderate/Probable): Level 2	RTV (doses of 50 mg and 100 mg) may inhibit CYP3A4, resulting in an increase in venetoclax C_max_ 2.3 to 2.4 times compared with venetoclax alone and AUC 6.1 and 8.1 times, respectively. After completing the gradual increase in the dose, venetoclax dose reductions of at least 75% are recommended when administered concomitantly with strong CYP3A inhibitors to maintain venetoclax exposures within the therapeutic range established for the treatment of chronic lymphocytic leukemia.
Vimblastine [49]	ATZ/RTV	(Grave/Possible): Level 2	RTV-boosted PIs may inhibit CYP3A4, resulting in an increase in vimblastine plasma levels, which increases the risk of severe hypokalemia (1.92 mEq/L) with electrocardiography fluctuations. Thus, this combination should be avoided; additionally, patients can be switched from RTV-boosted PI therapies to RAL, DTG, or BIC-based regimens.
**Antiparasitic/anthelmintic**			
Praziquantel [50]	EFV	(Moderate/Defined): Level 2	EFV may decrease AUC praziquantel by four-fold, increasing the risk of treatment failure. To monitor praziquantel effectiveness parameters, a dose adjustment may be necessary, but not exceeding the maximum.
**Antiplatelet agent**			
Clopidogrel [13,15,34,51,52,53]	ATV/RTV, DRV/RTV, LPV/RTV	(Grave/Defined): Level 1	PIs boosted with both RTV or COBI inhibit the bioactivation of clopidogrel to its active metabolite. Thus, these boosters may decrease C_max_ and AUC_0–4h_ clopidogrel active metabolite from 48 to 68% and from 51 to 69%, respectively, significantly reducing the antiplatelet effect of clopidogrel and increasing the risk for atherothrombotic events, including the recurrence of acute coronary syndrome. Clopidogrel should not be used as an antiplatelet agent for patients on boosted ARV therapies. Prasugrel as antiplatelet agent or unboosted regimens may be used in this context.
Ticagrelor [13,15,35]	ATV/RTV, DRV/RTV, LPV/RTV	(Grave/Possible): Level 2	PIs boosted with RTV may increase ticagrelor plasma levels, increasing the risk of bleeding. The use of ticagrelor with boosted PIs is not recommended. Other antiplatelet agents are suggested as an alternative, for instance, prasugrel.
Ticagrelor [15,53]	EFV	(Grave/Possible): Level 2	EFV-based ARV therapy may decrease ticagrelor plasma levels, increasing the risk of the recurrence of acute coronary syndrome. The use of ticagrelor with the EFV-based regimen is not recommended. Other antiplatelet agents are suggested as an alternative, for instance, prasugrel.
**Antiulcer/anti-H_2_**			
Famotidine [13,35,40,54]	RPV	(Moderate/Defined): Level 2	The bioavailability of RPV is pH-dependent; thus, famotidine through increasing the gastric pH may decreased RPV AUC concentrations by 76% when administered 2 h prior to RPV, increasing the risk of virological failure and the development of resistance. This combination may be used if famotidine is administered 4 h after or 12 h before RPV.
**Antiulcer/proton pump inhibitor**			
Lansoprazole [13,14,35,40,41]	RPV	(Grave/Defined): Level 1	The bioavailability of RPV is pH-dependent; thus, proton pump inhibitors may increase gastrointestinal pH and affect RPV absorption by decreasing its plasma levels. For instance, RPV administered with 20 mg omeprazole once daily decreased RPV AUC by 40% (if RPV was administered at a supratherapeutic daily dose of 150 mg). Thereby, oral RPV is contraindicated with proton pump inhibitors, and this combination should be avoided. However, spacing the administration of esomeprazole by 10 h could be used with RPV.
Omeprazole [13,14,35,40,41,42]			
Pantoprazole [13,14,35,40,41]			
Rabeprazole [13,14,35,40,41]			
**Atypical antipsychotic**			
Quetiapine [55]	ATZ/TRV DRV/RTV LPV/RTV	(Moderate/Probable): Level 2	Boosted PIs increase quetiapine plasma levels, mainly explained through CYP3A4 inhibition, which could lead to excess sedation or coma. The administration of quetiapine with PI-based regimens should be avoided or a quetiapine dose reduction is needing, mainly when a PI-based regimen is initiated in a patient on stable quetiapine therapy (a six-fold dose reduction is recommended).
**Contraceptive**			
Levonorgestrel (subdermal implant contraceptive) [15,34,56,57,58]	EFV	(Grave/Defined): Level 1	EFV-based ART therapy may significantly reduce levonorgestrel plasma levels by 61%, and the contraceptive effect can be decreased. In addition, even with the doubling of the dose of the levonorgestrel implants, concentrations remained >30% lower. Therefore, levonorgestrel implant is not recommended for women receiving long-term treatment with EFV. The patient should be counselled to use a barrier method as a complementary method of planning or to seek a safer contraceptive alternative.
Etonogestrel (subdermal implant contraceptive) [15,59]	EFV	(Grave/Defined): Level 1	EFV-based ART therapy may significantly reduce etonogestrel plasma levels by 82% in women after 24 weeks of combined use. Therefore, etonogestrel implant is not recommended for women receiving long-term treatment with EFV-based regimen. The patient should be counselled to use a barrier method as a complementary method of planning or to seek a safer contraceptive alternative.
Etonogestrel/ethinylestradiol (vaginal ring) [15,60,61]	EFV	(Grave/Defined): Level 1	EFV-based ART therapy may significantly reduce both the etonogestrel and ethinylestradiol levels of the intravaginal ring administered contraceptive by 79–81% and 56–59, respectively, in women after 21 days of coadministration. This pharmacokinetic interaction is most notorious in patients with the CYP2B6 slow metabolizer genotype (levels decrease by 93% for etonogestrel and by 75% for ethinylestradiol). Therefore, etonogestrel/ethinylestradiol (vaginal ring) is not recommended for women receiving treatment with the EFV-based regimen. The patient should be counselled to use a barrier method as a complementary method of planning or to seek a safer contraceptive alternative.
Depot medroxyprogesterone intramuscular [62]	EFV	(Moderate/defined) Level 2	EFV-based regimens in HIV and tuberculosis coinfected women and receiving rifampicin and isoniazid for tuberculosis increased the medroxyprogesterone clearance, leading to a more frequent contraceptive dosing (from 12 weeks to 8–10 weeks).
Etonogestrel (oral and subdermal implant contraceptive) [63]	NVP	(Grave/Defined): Level 1	In women with the single-nucleotide polymorphism NR1I2 63396 C>T (in gen CYP2B6), the NVP-based ART treatment may reduce etonogestrel plasma levels (C_min_ decrease by 39% and AUC_0-24_ weeks by 37%), significantly decreasing the contraceptive effect. Thus, the etonogestrel implant is not recommended in women with single-nucleotide polymorphism NR1I2 63396 C>T (in gen CYP2B6) receiving long-term treatment with the NVP-based regimen. The patient should be informed of the need to use a barrier method as a complementary method of planning or to seek a safer contraceptive alternative.
**Direct-acting antivirals**			
Glecaprevir/pibrentasvir [15,22,64,65]	ATV ATV/RTV	(Grave/Defined): Level 1	ATZ and ATV/RTV increase glecaprevir/pibrentasvir plasma levels (until pibrentasvir AUC 14-fold and until glecaprevir AUC 6.5-fold), which may cause elevations in transaminases, jaundice, and severe hyperbilirubinemia. This increase may be mainly explained due to P-gp, BCRP and/or OATP1B1/3 inhibition due to RTV (also COBI)-boosted regimens. The administration of glecaprevir/pibrentasvir with ATV or ATZ/RTV regimens is contraindicated.
Glecaprevir/pibrentasvir [15,22,64,65]	DRV/RTV, LPV/RTV	(Moderate/Defined): Level 2	Boosted PIs increase glecaprevir/pibrentasvir plasma levels, mainly explained through P-gp, BCRP, and/or OATP1B1/3 inhibition. The administration of glecaprevir/pibrentasvir with boosted PI regimens is not recommended.
Glecaprevir/pibrentasvir [15,22,64,65]	EFV	(Moderate/Defined): Level 2	EFV may reduce glecaprevir/pibrentasvir plasma levels, CYP3A4, and CYP2B6. The administration of glecaprevir/pibrentasvir with EFV is not recommended (overall, it is recommended to avoid the coadministration of any newer-generation direct-acting antiviral with efavirenz due to decreased plasma levels and the possible loss of efficacy).
Elbasvir/grazoprevir [15]	NVP	(Grave/Possible): Level 2	NVP may decrease grazoprevir/elbasvir plasma levels, CYP3A4, and CYP2B6. Consider alternative ARV or HCV treatments. If coadministration is necessary, it is recommended to monitor for HCV treatment efficacy.
Sofosbuvir/velpatasvir/voxilaprevir [15,65,66,67]	ATV/RTV	(Grave/Defined): Level 1	ATV/RTV single dose increases voxilaprevir AUC by 331%. Concomitant administration between ATV/RTV and voxilaprevir-containing regimens is not recommended.
Sofosbuvir/velpatasvir/voxilaprevir [15,65,66,67]	EFV	(Moderate/Defined): Level 2	EFV may decrease velpatasvir plasma levels. Concomitant use of sofosbuvir/velpatasvir with EFV-containing regimens is not recommended due to a 50% reduction in velpatasvir AUC.
Elbasvir/grazoprevir [15,22,65,68]	ATV/RTV, LPV/RTV, DRV/RTV	(Grave/Defined): Level 1	Grazoprevir exposure, mainly through OATP1B1/3 inhibition, is noticeably increased with the coadministration of ATV/RTV, LPV/RTV, and DRV/RTV, with AUC_0–24_ increasing by 10.58, 12.86, and 7.50, respectively. Similarly, elbasvir exposure is increased with the coadministration of ATV/RTV, LPV/RTV, and DRV/RTV, with AUC_0–24_ increasing by 4.76, 3.71, and 1.66, respectively. Thus, the coadministration of elbasvir/grazoprevir with PI-boosted RTV regimens is contraindicated, due to a marked increase in grazoprevir exposure. Therefore, PI-RTV-boosted regimens should be not used in patients with HCV/HIV coinfection who are being treated with elbasvir/grazoprevir.
Ombitasvir/paritaprevir/RTV/dasabuvir [69]	DRV/RTV	(Moderate/Probable): Level 2	Ombitasvir/paritaprevir/ritonavir plus dasabuvir (2D/3D) may cause a significant reduction in DRV levels by 41%. Thus, DRV plasma levels measurement during this combination may be needed. Additionally, ATV should be administered in the morning together with 2D/3D therapy.
**Direct-acting oral anticoagulant**			
Apixaban [13,15,22,34,70,71,72]	ATV/RTV, DRV/RTV, LPV/RTV	(Grave/Possible): Level 2	RTV-boosted PIs regimens may increase apixaban plasma levels, mainly through P-gp/CYP3A4 inhibition, increasing the risk of bleeding. Avoid this combination in patients who require apixaban 2.5 mg twice daily; similarly, in patients who require apixaban 5 mg or 10 mg twice daily, reduce apixaban dose by 50% (overall, a dose of 2.5 mg daily or 2.5 mg twice daily is used). Additionally, consider switching PI-RTV-boosted regimens to BIC-, DTG-, RAL-, or RPV-based regimens.
Apixaban [15,22,70]	EFV, ETR, NVP	(Grave/Possible): Level 2	EFV, ETR, and NVP may decrease apixaban plasma levels, via CYP3A4 induction, increasing the risk of thrombotic events. The use of these combinations is not recommended; it is suggested to use other anticoagulants as an alternative.
Edoxaban [13,15,22,35,70,72]	ATV/RTV, DRV/RTV, LPV/RTV	(Grave/Possible): Level 2	RTV-boosted PIs regimens may increase edoxaban plasma levels, mainly through P-gp inhibition. It may be necessary to monitor edoxaban safety parameters. For deep venous thrombosis and pulmonary embolism, it is recommended to reduce the dose of edoxaban from 60 mg to 30 mg daily. However, for stroke prevention in nonvalvular atrial fibrillation indication, no dose adjustment is recommended.
Rivaroxaban [13,15,35,72,73,74]	ATV/RTV, DRV/RTV, LPV/RTV	(Grave/Probable): Level 1	RTV-boosted PIs regimens may increase the rivaroxaban AUC and C_max_ by 153% and 55%, respectively, and increase the risk of bleeding events, mainly through P-gp/CYP3A4 inhibition. It is not recommended to use these combinations; it is suggested to use other anticoagulants as an alternative or to consider switching PI-RTV-boosted regimens to BIC-, DTG-, RAL-, or RPV-based regimens.
**Drug substance misuse**			
Amphetamine and substituted amphetamines (methamphetamine, methylenedioxymethamphetamin—MDMA; ‘Ecstasy’) [14,75,76,77,78,79,80]	ATV/RTV, DRV/RTV, LPV/RTV	(Grave/Possible): Level 2	RTV-boosted (ATV, DRV, or LPV) regimens may inhibit amphetamine metabolism, mainly through RTV CYP2D6 inhibition, leading to increased plasma levels (until 10-fold higher than expected) and toxicity, including fatal serotonergic reaction. Advise patients about the risks of these combinations.
**Food/supplements**			
Garlic-containing products [81]	DRV/RTV	(Grave/Possible): Level 2	Significant dietary garlic consumption may cause subtherapeutic DRV levels, probably due to the induction of duodenal P-gp, which increases the RTV efflux, decreasing the bioavailability and levels of RTV associated with a lower facility to boost DRV levels. The subtherapeutic DRV levels increase the risk of viral rebound. Thus, garlic consumption should be avoided during DRV-based antiretroviral regimen.
**Mineralocorticoid**			
Eplerenone [13,15,82,83]	ATV/RTV, DRV/RTV, LPV/RTV	(Grave/Possible): Level 2	RTV-boosted PIs regimens may increase eplerenone plasma levels, increasing the risk of possible life-threatening hyperkalemia. The use of this combination is not recommended.
**Special conditions**			
Adults > 65 years [84]	DRV and ATV	Moderate/probable	Higher steady-state plasma levels of DRV and ATV are found in PLHIV aged ≥ 65 years of age, compared to controls ≤ 49 years of age. Thus, in patients 65 years of age or older, the monitoring of plasma levels and dose adjustment of DRV and ATV are recommended.
Methadone for heroin addiction [85,86]	ATV/RTV DRV/RTV, LPV/RTV	(Moderate/Probable): Level 2	RTV-boosted PIs decrease (mainly through CYP2B6 induction) methadone plasma levels. The administration of methadone with RTV-boosted PI regimens need to increase the dose of MTD (mg/kg-body weight) by 50%.
Pregnancy [15,87,88]	ATV, DRV, LPV	(Grave/Possible): Level 2	Pregnancy may decrease PI exposure (the average AUC is decreased in a range of 5–56% during the first, second, and third trimesters vs. postpartum). Thus, RTV-boosted PIs regimens are needed in women who become pregnant. Overall, the comparisons between RTV-boosted PIs regimens showed no differences, for instance, there are no differences between ATV/RTV, LPV/TRV, or DRV/RTV regimens.
Pregnancy [15,87]	DOR	(Grave/Possible): Level 2	Pregnancy may decrease DOR exposure. Currently, its use is not recommended in women who become pregnant while on ART treatment.
Renal transplant recipients [89,90]	ATV/RTV	(Grave/Probable): Level 1	Regimen switch from ATV/RTV to DTG/FTC/TAF in a postrenal transplant recipient may cause subtherapeutic tacrolimus levels higher than expected from dolutegravir initiation (via organ cation transporter 2 inhibition), increasing the risk of increased serum creatinine and graft rejection. Thus, postrenal transplant recipients switched from ATV/RTV to DTG regimens need a tacrolimus dose increase (which could be up to 70-fold: 0.5 mg/week to 5 mg twice daily) to achieve the tacrolimus therapeutic range (usually, 4–8 ng/mL in postrenal transplantation) with careful clinical monitoring.
**Weight-loss drugs**			
Orlistat [91,92]	ATZ/RTV	(Grave/Possible): Level 2	Overall, the absorption of class II drugs (extensive presystemic metabolism, low water solubility, and high lipid permeability), for instance, atazanavir, is likely to be meaningfully affected by orlistat. Therefore, ATV/RTV-based regimen effectiveness may be affected by orlistat, which could be linked to the risk of virologic failure, loss of control of HIV viremia, and HIV viral rebound. Thus, this combination should be avoided.
Orlistat [91,92,93]	EFV	(Grave/Possible): Level 2	Overall, the absorption of class II drugs (extensive presystemic metabolism, low water solubility, and high lipid permeability), for instance, EFV, is likely to be meaningfully affected by orlistat. Therefore, EFV-based regimen effectiveness may be affected by orlistat, which could be linked to the risk of virologic failure, loss of control of HIV viremia, and HIV viral rebound. Thus, this combination should be avoided.

ART: antiretroviral therapy; ATV: atazanavir; ATV/RTV: atazanavir/ritonavir; AUC: area under the curve; BCRP: breast cancer resistance protein; C_max_: maximum concentration; C_min_: minimum concentration; CYP: cytochrome; DRV/COBI: darunavir/cobicistat; DRV/RTV: darunavir/ritonavir; DOR: doravirine; ECG: electrocardiogram; EFV: efavirenz; ETR: etravirine; HIV: human immunodeficiency virus; LPV/RTV: lopinavir/ritonavir; NNRTIs: non-nucleoside reverse transcriptase inhibitors; NVP: nevirapine; OATP: organic anion transporting polypeptide; P-gp: P-glycoprotein; PIs: protease inhibitors; RPV: rilpivirine; RTV: ritonavir.

**Table 4:** Drug interaction pairs with nucleoside/nucleotide reverse transcriptase inhibitors, integrase inhibitors, and cobicistat-boosted regimes, levels 1 and 2, systematically reported for the first time.

Pharmacological Group or Drugs Affected	Antiretroviral Agent	Gravity/Probability	Comments/Suggestions
**Anesthetic/anxiolytic/benzodiazepine**			
Midazolam (oral) [13,14,15,94]	EVG/COBI	(Grave/Defined): Level 1	COBI may reduce midazolam clearance near to 96%, increasing midazolam plasma levels, increasing the probability of respiratory depression, sedation, and muscle weakness. Thus, the use of this combination is not recommended. If necessary, parenteral midazolam should be used with close clinical monitoring or safer alternatives, such as lorazepam or propofol.
Alprazolam, clonazepam diazepam, and triazolam [13,14,15]		(Grave/Possible): Level 2	EVG/COBI may reduce the clearance of alprazolam, clonazepam, diazepam, and triazolam, increasing plasma levels and the probability of these benzodiazepines’ toxicity (respiratory depression, sedation, and muscle weakness). These combinations should be used with caution, monitoring for adverse effects of benzodiazepines. Additionally, safer alternatives, such as lorazepam, oxazepam, or temazepam, should be used.
**Cation-containing antacids/supplements and iron or zinc products**			
Aluminum hydroxide [13,14,15,22,95]	EVG/COBI, RAL, DTG, BIC, CAB	(Moderate/Defined): Level 2	Calcium, aluminum, and magnesium (Ca^2+^, Al^3+^, Mg^2+^)-containing antacids/supplements and also iron or zinc (Fe^2+^, Zn^2+^)-containing products may form chelates with InSTIs, thereby decreasing their absorption and efficacy. For instance, calcium carbonate may cause a reduction in both DTG and BIC AUC and C_max_ by up to 33–42%; ferrous fumarate may cause a decrease in AUC and C_max_ by 55–58% for DTG and by 63–70% for BIC. Additionally, BIC coadministered simultaneously with calcium, aluminum, and magnesium antacids under fasted conditions significantly reduced BIC exposure by 63–79%. In this sense: EVG/COBI: Should be administered at least 4 h separate from antacids or iron/zinc supplements. RAL: Do not coadminister with aluminum- and magnesium-containing antacids. Coadministration is possible with calcium-containing antacids, but only with 400 mg twice daily. Administer separately by 4 h from iron/zinc supplement administration. DTG: Administer at least 2 h before or at least 6 h after the administration of antacids containing calcium, magnesium, or aluminum. BIC: With antacids containing calcium should be administered together with food; do not coadminister simultaneously on an empty stomach. CAB: Administer at least 2 h before or at least 4 h after the administration of antacids containing calcium, magnesium, or aluminum. Additionally, it is very important to assess for use of over-the-counter drugs and vitamins/supplements in patients on InSTI-based antiretroviral therapy.
Calcium carbonate [13,14,15,22,95]			
Ferrous fumarate [13,14,15,22,95]			
Magnesium hydroxide [13,14,15,22,95]			
Zinc gluconate [13,14,15,22,95]			
**Antianginal**			
Ranolazine [15,102]	DRV/COBI	(Grave/possible): Level 2	DRV/COBI regimens may increase ranolazine plasma levels, mainly through CYP3A4 inhibition, increasing risk of adverse events (persistent episodes of nausea, vomiting, dyspepsia, anorexia, dizziness, syncope, and, potentially, auriculo-ventricular block). Thus, these combinations are not recommended.
**Antiarrhythmic**			
Dofetilide [14,15]	BIC DTG	(Grave/possible): Level 2	BIC and DTG administration may increase dofetilide plasma levels, for instance, 70–90% by DTG. Thus, these combinations are not recommended.
**Antiasthmatic/glucocorticoid**			
Fluticasone [13,14,35,103,104,105,106,107,108,109,110]	EVG/COBI	Grave/Probable): Level 1	EVG/COBI causes a significant increase in exposure to fluticasone (due to CIP3A4 inhibition), which can cause Cushing’s syndrome and adrenocortical suppression, leading to secondary adrenal insufficiency. This drug interaction can occur after a brief exposure (10–14 days) and with fluticasone nasal drops. This combination should be avoided. If it is needed, beclomethasone can be used as an alternative, but with caution. Additionally, similar to the RTV-based regimen, consider switching COBI-boosted regimen to BIC-, DTG-, RAL-, or RPV-based regimens.
Budesonide [13,14,35,103,104,105]	EVG/COBI	Grave/Possible): Level 2	EVG/COBI causes a significant increase in exposure to budesonide (due to CIP3A4 inhibition), which can cause Cushing’s syndrome and adrenocortical suppression, leading to secondary adrenal insufficiency. This combination should be used cautiously, monitoring budesonide toxicity (in acutely unwell, hypotensive, and hyponatremic patients).
Mometasone [13,14,35,103,104,108]	EVG/COBI	(Grave/Possible): Level 2	EVG/COBI may increase mometasone plasma levels (due to CIP3A4 inhibition), which can cause Cushing’s syndrome and adrenocortical suppression, leading to secondary adrenal insufficiency. This combination should be used cautiously, monitoring mometasone toxicity (in acutely unwell, hypotensive, and hyponatremic patients).
Triamcinolone (injectable) [13,35,103,108,111,112,113]	EVG/COBI	(Grave/Probable): Level 1	EVG/COBI causes a significantly increase in exposure to triamcinolone (due to CIP3A4 inhibition), which can cause Cushing’s syndrome and adrenocortical suppression, leading to secondary adrenal insufficiency. This combination should be avoided. If it is needed, methylprednisolone can be used as an alternative, but with caution. Additionally, similar to the RTV-based regimen, consider switching COBI-boosted regimen to BIC-, DTG-, RAL-, or RPV-based regimens.
Triamcinolone (intrabursal) [13,15,35,108,114]	DRV/COBI	(Grave/possible): Level 2	DRV/COBI may increase triamcinolone plasma levels (due to CIP3A4 inhibition), which can cause Cushingoid features and an undetectable cortisol, leading to secondary hypoadrenalism. This combination should be avoided. Additionally, similar to the RTV-based regimen, consider switching COBI-boosted regimen to BIC-, DTG-, RAL-, or RPV-based regimens.
Betamethasone (topical) [15,115]	DRV/COBI	(Grave/possible): Level 2	DRV/COBI may increase betamethasone plasma levels (due to CIP3A4 inhibition), if it is used topically and for ≥2 months. This alteration may cause Cushingoid features and an undetectable cortisol, leading to secondary hypoadrenalism. In patients on COBI-based regimen, the use of chronic topical betamethasone should be avoided.
**Anticoagulant/antivitamin K**			
Warfarin [116]	DTG	(Grave/Possible): Level 2	Switching from NEV-based regimen to DTG-based regimen may increase warfarin plasma levels and INR. Close monitoring of INR is mandatory, due to warfarin dosing requirements potentially significantly reducing.
Warfarin [15,35,70,117]	EVG/COBI	(Grave/possible): Level 2	EVG/COBI appears to have a strong induction on CYP2C9, leading to a decrease in warfarin plasma levels and INR and a warfarin dosage increase.
Warfarin [118]	EVG/COBI/TAF/FTC	(Grave/possible): Level 2	EVG/COBI/TAF/FTC, mainly TAF, may increase warfarin plasma levels and INR from 2.7 to 4.5 (after 7 days) using the usual warfarin dosage, then to 6.5 (after 14 days), despite a reduction in the warfarin dose. This effect could be attributed to TAF; however, the mechanism is not clear.
Warfarin [13,14,35,119,120]	DRV/COBI	(Moderate/Probable: Level 2)	DRV/COBI may increase warfarin plasma levels, increasing the risk of bleeding. However, boosted ARVs may cause both inhibiting/inducing effects on CYPs and, therefore, are expected to alter warfarin plasma levels. Therefore, close monitoring of INR to determine appropriate warfarin dose in patients on ARV treatment is recommended.
**Anticonvulsant**			
Carbamazepine–oxcarbazepine [14,15,18,35,96]	DTG	(Moderate/Probable): Level 2	Carbamazepine or oxcarbazepine may cause a significantly lower trough concentration of DTG by 83% (191 ± 78 vs. 1096 ± 510 ng/mL), and may be associated with the induction of CYP3A4 and UGT1A1 (metabolizing enzymes). Thus, these combinations should be avoided, owing to the potential risk of virological failure. Additionally, in ART-naive or ART-experienced (except for InSTIs-naive) patients, increase DTG to 50 mg twice daily use. However, in InSTIs-experienced patients with known or suspected InSTIs resistance, this combination should not be used. Additionally, alternatives, such as levetiracetam and/or gabapentin (not CYP metabolized), may be considered.
Phenobarbital [14,15,35,121]	DTG	(Moderate/Probable): Level 2	Phenobarbital may cause a significantly lower trough concentration of DTG by 83% (191 ± 78 vs. 1096 ± 510 ng/mL), and may be associated with the induction of CYP3A4 and UGT1A1 (metabolizing enzymes). Thus, these combinations should be avoided, owing to the potential risk of virological failure. Additionally, in ART-naive or ART-experienced (except for InSTIs-naive) patients, increase DTG to 50 mg twice daily use. However, in InSTIs-experienced patients with known or suspected InSTIs resistance, this combination should not be used. Additionally, alternatives, such as levetiracetam and/or gabapentin (not CYP metabolized), may be considered.
Valproic acid [122,123,124,125]	DTG	(Moderate/Probable): Level 2	Valproic acid may decrease DTG plasma levels due to a mechanism, including protein displacement, increasing the induction of CYP3A4 and UGT1A1 (metabolizing enzymes) or P-glycoprotein (transporting enzyme). Closely monitor DTG effectiveness parameters, because it may lead to increased resistance and increased viral load. In addition, other anticonvulsant alternatives such as levetiracetam are suggested. However, if both dolutegravir is taken with food and patient is adherent to treatment, this DDI may be not clinically relevant. More clinical information is needed.
Atovaquone/proguanil [15,20,21,22,24,25]	ATZ/RTV LPV/RTV	(Moderate/Defined): Level 2	ATZ/RTV- or LPV/RTV-based antiretroviral therapy decreases AUC atovaquone/proguanil (with ATV/RTV: atovaquone AUC 46% and proguanil AUC by 41%; with LPV/TRV: atovaquone AUC by 74%), leading to possible atovaquone/proguanil prophylaxis failures in HIV-infected patients. Thus, these combinations should be used with caution or avoided. Additionally, an increase in the dose of atovaquone/proguanil or other alternative drug for malaria prophylaxis may be considered.
**Antimicrobial/sulfonamide**			
Cotrimoxazole (trimethoprim/sulfamethoxazole [21,126]	3TC	(Moderate/Defined): Level 2	Trimethoprim administration may cause 43% increase in AUC and a 35% decrease in renal clearance of 3TC, leading to increase in the plasma levels and in the risk to development metabolic acidosis and hyperlactatemia. The dose of 3TC may be reduced in patients with renal disease; however, in patients with normal renal function, there is no need to adjust the dose.
**Antimicrobial/antituberculosis**			
Rifampicin [13,15,34,94,123,124]	BIC	(Grave/Defined): Level 1	Rifampicin may induce BIC metabolism, causing a significant decrease in AUC (60%) and in trough concentrations (80%), resulting in a loss of therapeutic effect. Dose adjustment recommendations for coadministration of BIC and rifampicin have not been established; however, the combination is contraindicated.
Rifabutin [13,15,35,127,129]	BIC	(Moderate/Defined): Level 2	Rifabutin may induce BIC metabolism, causing a significant decrease in AUC (38%) and trough concentrations (56%), and might generate ineffectiveness. Dose adjustment recommendations for coadministration of BIC and rifabutin have not been established and the combination is not recommended.
Rifampicin [15,35,40,96,130,131]	CAB	(Grave/Defined): Level 1	Rifampicin may induce CAB metabolism, causing a decrease in the CAB AUC and the half-life by 59% and 57%, respectively, while clearance is increased 2.4-fold, resulting in loss of therapeutic effect. Additionally, oral rifampicin may decrease exposure to intramuscular CAB by 44%. Dose recommendations for the coadministration of CAB and rifampin have not been established; however, the combination is not recommended, regardless of the route of CAB administration.
**Antimigraine**			
Ergotamine [15,132,133]	EVG/COBI	(Grave/Possible): Level 2	Similar to RTV, COBI may increase plasma ergotamine concentrations by inhibiting CYP3A4, increasing the probability of ergotism, including gastrointestinal and muscle disorders, as well as the sudden onset of severe pain and paresthesias in both legs associated with acute ischemia. To monitor ergotamine safety parameters, it is important to counsel about the risk associated with the use of ergotamine-containing drugs through self-medication.
**Antineoplastic**			
Vinblastine [134]	AZT	(Grave/Possible): Level 2	AZT may increase the probability of hematologic toxicity with the use of vinblastine. This combination is not recommended due to the possibility of serious adverse effects.
Vinblastine [108,135]	EVG/COBI	(Grave/Possible): Level 2	COBI-boosted regimens may increase vimblastine plasma levels, increasing the risk of peripheral neuropathy (sensory–motor lower and upper limbs). The use of vimblastine with boosted ARV (including both RTV and COBI) is not recommended. Unboosted ARV regimens may be used in this context.
**Antiplatelet agents**			
Clopidogrel [13,35,51,52]	EVG/COBI	(Grave/Defined): Level 1	COBI-boosted regimens inhibits the bioactivation of clopidogrel to its active metabolite, significantly reducing the antiplatelet effect of clopidogrel and increasing the risk for atherothrombotic events. Clopidogrel should not be used as an antiplatelet agent for patients on EVG/COBI-based regimens. Prasugrel as an antiplatelet agent or unboosted regimens may be used in this context.
Ticagrelor [13,15,35]	EVG/COBI	(Grave/possible): Level 2	EVG/COBI may increase ticagrelor plasma levels, increasing the risk of bleeding. The use of ticagrelor with boosted ARVs is not recommended. Other antiplatelet agents are suggested as an alternative.
**Contraceptive**			
Drospirenone/ethinyl estradiol [136]	ATV/COBI DRV/COBI	(Moderate/probable): Level 2	Drospirenone AUC is increased with the coadministration of COBI-containing regimens (1.6-fold with DRV/COBI and 2.3-fold with ATV/COBI), maybe due to COBI-mediated CYP3A inhibition. The increase in drospirenone AUC increases the risk of drospirenone-associated hyperkalemia. Therefore, with DRV+COBI monitoring for hyperkalemia is recommended, and the combination of ATV+COBI with drospirenone should be avoided.
**Direct-acting antivirals**			
Elbasvir/grazoprevir [15,22,65,84]	EVG/COBI	(Moderate/Defined): Level 2	Elbasvir and grazoprevir exposure is increased with the coadministration of EVG/COBI; thus, AUC_0–24_ increases by 2.2 and 5.4, respectively. Due to the increased grazoprevir exposure associated with the coadministration of EVG/COBI, this combination is not recommended in patients with HCV/HIV coinfection, who are being treated with elbasvir/grazoprevir.
**Direct oral anticoagulant**			
Apixaban [13,15,22,35,70,71,72]	EVG/COBI	(Grave/Possible): Level 2	EVG/COBI may increase apixaban plasma levels mainly through P-gp/CYP3A4 inhibition. Avoid this combination in patients who require apixaban 2.5 mg twice daily; similarly, in patients who require apixaban 5 mg or 10 mg twice daily, reduce apixaban dose by 50%. Consider switching COBI-boosted regimens (similar to RTV- and RTV-boosted regimens) and PIs-RTV boosted regimens to BIC-, DTG-, RAL-, or RPV-based regimens.
Apixaban [13,15,22,35,70,71,72,137]	DRV/COBI	(Grave/Possible): Level 2	DRV/COBI may increase apixaban plasma levels mainly through P-gp/CYP3A4 inhibition. Avoid this combination in patients with a recent vascular procedure or in those who require apixaban 2.5 mg twice daily; similarly, in patients who require apixaban 5 mg or 10 mg twice daily, reduce apixaban dose by 50%. Consider switching COBI-boosted regimen (similar to RTV and RTV-boosted regimens) to BIC-, DTG-, RAL-, or RPV-based regimens.
Dabigatran [13,15,34,69,71,138,139,140]	EVG/COBI	(Grave/Defined): Level 1	COBI may increase AUC and C_max_ dabigatran by 127% in both, leading to an increase in thrombin time assessment by 33% for the area-under-the-effect curve, since time zero to 24 h and 51% at 24 h, mainly through P-gp inhibition. Overall avoid this combination. Consider switching COBI-boosted base regimen to BIC, DTG, RAL, RPV-, or inclusive to RTV-boosted regimen. Dabigatran dosing recommendation depends on estimated creatinine clearance (CL_Cr_): (a) Severe renal failure (CL_Cr_ < 30 mL/min): coadministration of dabigatran and COBI (and RTV) should be avoided. (b) Moderate renal failure (CL_Cr_ 30–59 mL/min): dabigatran and COBI: 75 mg twice daily (dabigatran and RTV: 110 mg twice daily). (c) Normal renal function (CL_Cr_ > 60 mL/min): dabigatran and COBI: 110 mg twice daily (dabigatran and RTV: 150 twice daily, usual dose).
Edoxaban [13,15,35,70,72]	ATV/COBI	(Grave/Possible): Level 2	ATV/COBI may increase edoxaban plasma levels mainly through P-gp inhibition. It is not recommended to use this combination; it is suggested to use other anticoagulants as an alternative or to switch COBI-boosted regimen (similar to RTV- and RTV-boosted regimens) to BIC-, DTG-, RAL-, or RPV- based regimens.
Edoxaban [13,15,22,35,70,72]	DRV/COBI	(Grave/Possible): Level 2	DRV/COBI may increase plasma concentrations of edoxaban through the inhibition of CYP3A4 and P-gp. It is recommended to monitor edoxaban safety parameters, especially in patients with renal disease.
Edoxaban [13,15,22,35,71,72]	EVG/COBI	(Grave/Possible): Level 2	EVG/COBI may increase edoxaban plasma levels through the inhibition of CYP3A4 and P-gp. It is recommended to monitor edoxaban safety parameters. It is recommended to reduce the dose of edoxaban from 60 mg to 30 mg in patients with deep venous thrombosis and pulmonary embolism.
Rivaroxaban [13,15,35,73,74,141]	EVG/COBI	(Grave/Possible): Level 2	EVG/COBI may increase rivaroxaban plasma levels and bleeding events mainly through P-gp/CYP3A4 inhibition. It is not recommended to use this combination; it is suggested to use other anticoagulants as an alternative or to switch COBI-boosted regimen (similar to RTV- and RTV-boosted regimens) to BIC-, DTG-, RAL-, or RPV-based regimens.
**Drug substance misuse**			
Amphetamine and substituted amphetamines (methamphetamine, methylenedioxymethamphetamine—MDMA; ‘Ecstasy’) [14,15,75,76,80,141,142]	EVG/COBI	(Grave/Possible): Level 2	EVG/COBI (and other COBI-boosted regimens), mainly through CYP2D6 inhibition, may increase plasma levels of amphetamines, prolong their clinical effects, and increase toxicity. Overall, concomitant use of CYP2D6 inhibitors such as COBI (RTV) could increase amphetamine toxicity due to drug accumulation. In these cases, it is recommended to use unboosted regimens, and counselling patients about the risks of these combinations.
Ketamine [14,15,75,76,142,143]	EVG/COBI DRV/COBI	(Grave/Possible): Level 2	EVG/COBI (and other boosted regimens), mainly through CYP3A4 inhibition may increase plasma levels of ketamine, prolong its clinical effects, and increase toxicity. Overall, the concomitant use of CYP3A4 inhibitors, such as COBI (RTV), could increase ketamine toxicity due to drug accumulation. In these cases, it is recommended to use unboosted regimens and counselling patients about the risks of these combinations.
**Feminizing hormone therapy (FHT)**			
Estradiol valerate/cyproterone acetate [144,145,146,147,148,149,150,151,152]	TDF/FTC (on- demand PrEP: 2, 1, 1)	(Moderate/Probable): Level 2	TDF/FTC exposure may be reduced by 12–32% among transgender women vs. cisgender men in the presence of FHT, suggesting that it may potentially affect the effectiveness of PrEP. Additionally, active metabolites and competing deoxynucleotides in the rectal tissue of transgender women are seven-fold lower in rectal TFVdp:dATP vs. cisgender men. However, some results show no interaction for FHT on tenofovir levels and supporting PrEP use among transgender women using FHT. The clinical significance and the mechanism of this drug interaction is still unclear and this reduction may impact the HIV protective efficacy of a daily PrEP regimen. However, FHT may produce concentrations too low for the consistent prevention of HIV infection on demand in the PrEP regimen: 600 mg TDF/400 mg FTC on day 1 and 300 mg TDF/200 mg FTC on days 2 and 3.
**Foods/supplements**			
Guggulsterone-containing supplements [153]	EVG/COBI	(Grave/Possible): Level 2	Guggulsterone may be a pregnane X receptor (PXR) agonist, causing the induction of the expression of CYP3A genes. Thus, the concomitant administration of guggulsterone-containing supplements may cause a significant reduction in the EVG plasma levels, increasing the risk of viral rebound after a 4-month treatment. Thus, guggulsterone-containing thermogenic complex consumption should be avoided during the EVG/COBI treatment.
Sorbitol [154]	3TC	(Grave/Possible): Level 2	Sorbitol 3.2, 10.2, and 13.4 g decrease 3TC C_max_ by 28%, 52%, and 55% and AUC_0–24_ by 20%, 39%, and 44%, respectively, explained due to an absorption-based interaction, which can lead to 3TC-based regimen therapeutic failure. Thus, the coadministration of 3TC-based regimens with sorbitol-containing medicines should be avoided; however, if it is coadministered, monitor the virologic response.
**Mineralocorticoid**			
Eplerenone [13,15,83]	EVG/COBI	(Grave/Possible): Level 2	EVG/COBI may increase eplerenone plasma levels, by affecting the hepatic/intestinal enzyme CYP3A4 metabolism, increasing the risk of hyperkalemia. The use of this combination is not recommended.
**Phosphodiesterase 5 inhibitors**			
Sildenafil [13,14,15,155]	EVG/COBI DRV/COBI	(Grave/Possible): Level 2	COBI-boosted (similar to RTV-boosted) regimens may increase sildenafil plasma levels (10 mg 3 times daily causes concentrations similar to 20 mg 3 times daily, recommended dose for pulmonary arterial hypertension), mainly through CYP3A4 inhibition. Therefore, this combination increases the risk of hypotension and respiratory failure and it is not recommended. Failing that, begin sildenafil at 10 mg 3 times daily (lower doses) and monitor its plasma levels.
**Special conditions**			
Pregnancy [15,156]	BIC	(Grave/Possible): Level 2	Pregnancy may decrease BIC exposure. More research and information regarding clinical efficacy and safety of BIC during pregnancy is needed. This drug is not currently recommended in pregnancy.
Pregnancy [15,87,157,158]	EVG/COBI	(Grave/Defined): Level 1	Overall, COBI, mainly with the EVG regimen, should be avoided in pregnancy due to lower concentrations of COBI and its boosted drugs—EVG, DRV, and ATV (the average AUC is decreased in a range of 5–56% during the first, second, and third trimester vs. postpartum). For instance, pregnancy may decrease the AUC of EVG and COBI by 24% and 44%, respectively, during the second trimester, increasing the risk of virologic failure and mother-to-child transmission. Additionally, pregnancy may decrease the AUC of COBI by 59% during the third trimester. If women become pregnant during treatment with EVG/COBI, it should be changed to an alternative boosting, such as ritonavir, or the viral load should be monitored every month.
Pregnancy [15,131,159]	CAB/RPV	(Grave/Possible): Level 2	CAB/RPV is not recommended for use in pregnancy, due to lack in data existing for people who are trying to conceive or who become pregnant. Overall, CAB is not recommended during pregnancy and there is a need for additional studies regarding the safety of this drug during pregnancy.
Pregnancy [87,160,161]	TDF monotherapy	(Moderate/Defined): Level 2	Among women with HIV on TDF with RTV/PIs or without RTV/PIs and women with HBV on treatment with TDF monotherapy, TFV exposure is decreased by nearly 25% during the third trimester of pregnancy, probably associated with an increase because of an increased volume of distribution and renal excretion. However, this does not apply to LPV/RTV, due to some results having showed that tenofovir exposure is comparable between the third trimester of pregnancy and postpartum among women receiving concomitant LPV/RTV.
Renal transplant recipients [162]	EVG/COBI	Grave/possible): Level 2	EVG/COBI may cause supratherapeutic tacrolimus concentrations in postrenal transplant recipients (level of 111.2 ng/mL at 1 week, goal trough level 4–6 ng/mL), mainly via CYP3A inhibition, increasing risk of acute kidney damage (serum creatinine level increasing from 1.5 to 2.3 mg/dL). Thus, in postrenal transplant recipients, this combination should be avoided.
**Statins**			
Lovastatin [13,14,15,22,35,163]	EVG/COBI	(Grave/Possible): Level 2	EVG/COBI may increase lovastatin plasma levels, increasing the risk of muscle toxicity, severe muscle pain, rhabdomyolysis, and acute renal injury. This combination is not recommended. Safer alternatives, such as pravastatin or fluvastatin, should be used.
Simvastatin [13,14,15,22,35,163,164]	EVG/COBI	(Grave/Possible): Level 2	EVG/COBI may increase simvastatin plasma levels, increasing the risk of muscle toxicity, severe muscle pain, rhabdomyolysis, and acute renal injury. This combination is not recommended. Safer alternatives, such as pravastatin or fluvastatin, should be used.
Pravastatin/fenofibrate [14,15,22,165]	EVG/COBI	(Grave/Possible): Level 2	EVG/COBI may increase pravastatin plasma levels, increasing the risk of muscle toxicity, severe muscle pain, rhabdomyolysis, and acute renal injury. It is possible that COBI increases pravastatin plasma levels through complex mechanisms, including inhibition of BCRP intestinal and biliary efflux pumps; the inhibition of the P-gp intestinal, biliary, and renal efflux pumps; and the inhibition of OATP1B1-mediated entry into hepatocytes. This combination should be used with caution, using the lowest effective pravastatin dose together with monitoring for muscle toxicity signs.
Atorvastatin [13,14,15,22,35,83,163,166]	EVG/COBI	(Grave/Possible): Level 2	ATV/COBI and DRV/COBI may increase atorvastatin AUC by 822–853% and 292%, respectively, increasing the risk of muscle toxicity, severe muscle pain, rhabdomyolysis, and acute renal injury. These combinations should be used with caution, atorvastatin doses should not exceed 20 mg daily (10 mg with ATV/COBI), and monitor for typical adverse effects associated with statins. Additionally, safer alternatives, such as pravastatin or fluvastatin, should be used.
Rosuvastatin [13,14,15,22,35,83,163,166]	ATV/COBI DRV/COBI	(Moderate/Defined): Level 2	ATV/COBI and DRV/COBI may increase rosuvastatin AUC by 218–242% and 89–93%, respectively, increasing the risk of muscle toxicity, severe muscle pain, rhabdomyolysis, and acute renal injury. These combinations should be used with caution, atorvastatin doses should not exceed 20 mg daily (10 mg with ATV/COBI), and monitor for typical adverse effects associated with statins. Additionally, safer alternatives, such as pravastatin or fluvastatin, should be used.
Simvastatin [13,14,15,22,35,166]	ATV/COBI DRV/COBI	(Moderate/Defined): Level 2	DRV may increase simvastatin plasma levels, increasing the risk of muscle toxicity, severe muscle pain, rhabdomyolysis, and acute renal injury. This combination is not recommended. Safer alternatives, such as pravastatin or fluvastatin, should be used.
**Uricosuric agent**			
Probenecid [167]	TDF/FTC (on-demand PrEP: 2,1,1)	(Moderated/Defined): Level 2	Probenecid increases the AUC of TDF and FTC by 61% and 68%, respectively, mainly through the inhibition of OAT1/OAT3, which leads to decreased TDF uptake into renal cells available for elimination. However, the TDF diphosphate (TDF active form) concentrations in peripheral blood mononuclear cells are higher (~30%) at 24 h, but then fall significantly lower by 40% at 72 h. In patients on-demand PrEP with TDF/FTC (2, 1, 1), the risk/benefit of probenecid addition should be assessed.
		

ARV: antiretroviral; ATV/COBI: atazanavir/cobicistat; AUC: area under the curve; AZT: zidovudine; BCRP: breast cancer resistant protein; BIC: bictegravir; BCRP: breast cancer resistance protein; CAB: cabotegravir; COBI: cobicistat; CYP: cytochrome; DRV/COBI: darunavir/cobicistat; DTG: dolutegravir; ECG: electrocardiogram; EVG: elvitegravir; EVG/COBI: elvitegravir/cobicistat; EVG/COBI/TAF/FTC: elvitegravir/cobicistat/tenofovir alafenamide/emtricitabine; INR: international normalized ratio; InSTIs: integrase strand transfer inhibitors; MATE 1: toxin extrusion proteins and multiple drugs; NRTIs: nucleoside/nucleotide reverse transcriptase inhibitors; OAT1/OAT3: organic anion transporters; OCT2: organic cation transporters; P-gp: P-glycoprotein; PrEP: pre-exposure prophylaxis; RAL: raltegravir; TDF: tenofovir disoproxil-fumarate; TDF/FTC: tenofovir disoproxil-fumarate/emtricitabine; TDF/FTC/EVG/COBI: tenofovir disoproxil-fumarate/emtricitabine/elvitegravir/cobicistat; TDF/FTC/RAL: tenofovir disoproxil-fumarate/emtricitabine/raltegravir; UGT1A1: uridinadifosfatoglucoronosiltransferasa; 3TC: lamivudine.

**Table 5:** Drug interaction pairs with evidence of absence of clinically relevant drug interactions (Level 5) systematically reported for the first time.

Pharmacological Group or Drugs Affected	Antiretroviral Agent	Comments/Suggestions
**Anesthetic/anxiolytic/benzodiazepine**		
Midazolam [168]	CAB	Midazolam 3 mg once daily administered with CAB 30 mg once daily only increases midazolam AUC and C_max_ by 10% and 9%, respectively. Thus, CAB does not modify the pharmacokinetics of midazolam. It is a safe combination, therefore, does not require a dose adjustment.
**Antimalarial**		
Chloroquine [169]	EFV	Efavirenz-based ART and chloroquine 150 mg daily is well tolerated and EFV does not change chloroquine plasma levels. Thus, this combination is safe and it does not require a dose adjustment.
**Antimicrobial/antituberculosis**		
Cotrimoxazole (trimethoprim and sulfamethoxazole) [21,170]	NVP	No significant changes in plasma levels of NVP and clotrimazole are generated. This combination is safe and does not require a dose adjustment.
Levofloxacin [171]	EFV, NVP	NFV or EFV do not affect levofloxacin AUC and C_max_; additionally, there is no significance in the NVP or EFV plasma levels with and without levofloxacin. Therefore, these combinations are safe and dose adjustments are not needed.
Amikacin, ethambutol, ethionamide, pyrazinamide, terizidone [172]	LPV/RTV	Coadministration of antituberculosis drugs (high-dose isoniazid, pyrazinamide, ethambutol, ethionamide, terizidone, and amikacin) and LPV/RTV in HIV-infected children generates minor changes both in AUC and C_max_ of antituberculosis drugs and LPV/TRV. These combinations are safe and dose adjustments are not needed.
Rifabutin [21,173]	ATV/RTV	No significant changes in plasma levels of ATV/RTV (330/110 mg daily) and rifabutin (300 mg thrice-weekly or 150 mg daily doses) are generated. The combination of ATV/RTV (330/110 mg daily) and rifabutin (300 mg thrice weekly or 150 mg daily doses) is safe.
Rifabutin [21,127,174]	CAB (oral)	Rifabutin has a minor effect on AUC and C_max_ CAB, resulting in CAB plasma levels to maintain viral suppression in HIV-1-infected persons. Thus, rifabutin and oral CAB can be coadministered without dose adjustments.
Rifampicin [21,175,176]	DTG	DTG (50 mg twice daily) in patients on rifampicin-based treatment is effective and safe. Therefore, among ART-naive patients with HIV on rifampicin-based tuberculosis treatment, DTG-based ART (50 mg twice daily) is effective and safe.
**Attachment inhibitor**		
Fostemsavir [177,178,179]	ETR/DRV/RTV	Fostemsavir 600 mg twice daily combined with ETR 200 mg twice daily causes a decrease in AUC of temsavir (active drug) by 50%. However, when fostemsavir and ETR are combined with DRV/RTV 600/100 mg twice daily, the temsavir AUC increases by 34%. Thus, the coadministration of fostemsavir/etravirine/DRV/RTV 600/200/600/100 mg twice daily does not need a dose adjustment and it is effective and safe, which has been evidenced from phase IIb/III studies. Thus, this combination has been approved for patients with HIV not able to be treated with other options.
**Direct-acting antivirals**		
Elbasvir/grazoprevir [180]	DTG or RAL	The coadministration of elbasvir/grazoprevir with RAL or DTG does not produce relevant clinical drug interactions and it is overall well tolerated. These results support the use of these combinations without dose adjustments for elbasvir, grazoprevir, RAL, or DGV in HCV/HIV-coinfected persons.
Simeprevir [181]	DTG	Simeprevir causes a slight increase in the AUC by 15% of DTG; however, it is bioequivalent and DTG plasmatic levels are within the safely established therapeutic range. Thus, simeprevir and DTG can be safely coadministered without dose adjustments.
Ledipasvir/sofosbuvir [182]	FTC/RPV/TAF	Although the C_max_ and AUC of tenofovir (main metabolite of TAF) increase by 62% and 75%, respectively, the resulting absolute tenofovir exposures are noticeably inferior to the historical tenofovir exposures following TDF; as a consequence, this increase in tenofovir is not considered to be clinically relevant. Therefore, fixed-dose combinations of ledipasvir/sofosbuvir 90/400 can be coadministered with fixed-dose combinations of FTC/RPV/TAF 200/25/25 mg for HIV without the need for dosage adjustments.
**Disease**		
Mild to moderate hepatic impairment (a Child–Pugh score of 7–9) [157,183,184,185,186,187,188]	BIC, CABO, DTG, EVG/COBI, RAL	Moderate liver impairment does not affect plasma InSTIs exposure compared with persons without hepatic impairment. Thus, InSTIs may be administered without a dose adjustment in patients with moderate liver impairment.
Mild to severe renal impairment (estimated creatinine clearance <30–60 mL/min) [157,183,184,188,189,190,191,192]	BIC, CAB, DTG, EVG/COBI, RAL	Severe renal impairment does not affect plasma InSTIs exposure compared with persons without severe renal impairment. Thus, these drugs may be administered without a dose adjustment in patients with renal impairment, including patients on chronic hemodialysis (mainly for CAB and EVG/COBI). However, when TDF is coformulated with any InSTIs, its use may be limited due to TDF, caution should be taken in persons with renal insufficiency.
**Foods/supplements**		
Food (standard breakfast and protein-rich drink) [193,194,195]	EVG/COBI/FTC/TAF	EVG/COBI/FTC/TAF administration under fasted conditions is associated with a decrease by 50% and 57% in the mean AUC and C_max_ of EVG, respectively. Thus, EVG/COBI/FTC/TAF should be administered orally once daily with food, for instance, a standard breakfast (413 kcal, 10% fat: two slices of bread with strawberry jam, one boiled egg, and 160 g of grape juice). It is important to denote that oral EVG/COBI/FTC/TAF administration once daily with a nutritional protein-rich drink generates similar EVG/COBI exposure as administration with standard breakfast. Regardless, food or a nutritional protein-rich drink did not affect the bioavailability of EVG/COBI/FTC/TAF.
**Injectable contraceptive**		
Depot medroxyprogesterone [196]	TDF (1% gel)	TDF 1% vaginal gel administered topically for HIV-1 prevention in women resulted in high and protective mucosal concentrations with or without use of depot medroxyprogesterone (DMPA) or oral contraceptives. TDF diphosphate (intracellular active metabolite of TDF) reached higher vaginal tissue concentrations several times above estimated protective levels in women using DMPA. This combination is safe and does not require a dose adjustment.
**NNRTI**		
Etravirine [176,197]	CAB	In total, 30 mg of CAB for 10 days followed by the coadministration of ETR 200 mg twice daily for 14 days does not cause significantly changes in plasma levels for either drug. Thus, CAB may be administered with ETR without a dosage adjustment for either agent.
Etravirine [176,198,199]	RAL	ETR and RAL have no clinically significant pharmacokinetic interactions; therefore, ETR and RAL plasmatic levels are acceptable in most patients. The virologic efficacy and therapeutic success rates at week 48 are 99.4% and 94.5%, respectively; results are similar at week 96. Additionally, the combination is generally well tolerated (most drug-related adverse clinical experiences are minor and generally temporary in nature). Thus, etravirine 200 mg/RAL 400 mg twice daily is an effective and safe dual therapy. However, the coadministration of etravirine with RAL 1200 mg daily should be avoided due to a decrease in the C_min_ of RAL near to 34%.
**Oral contraceptive**		
Ethinylestradiol/Levonorgestrel [40,200]	CAB	There are no significant changes in plasma levels of CAB and ethinylestradiol/levonorgestrel containing oral contraceptive. Additionally, repeat doses of oral CAB do not have a significant effect on ethinylestradiol/levonorgestrel plasma levels or effects, which supports that this combination is safe and requires no dose adjustment.
Ethinylestradiol/Levonorgestrel [196]	TDF (1% gel)	TDF 1% vaginal gel administered topically for HIV-1 prevention in women resulted in high and protective mucosal concentrations with or without the use of ethinylestradiol/levonorgestrel as an oral contraceptive. This combination is safe and does not require a dose adjustment.
Ethinylestradiol/norgestimate [201,202]	TDF	TDF and norgestimate-ethinyl estradiol coadministration shows a lack of clinically significant drug interactions. TDF does not cause significant changes to norgestimate or ethinyl estradiol plasmatic levels; similarly, norgestimate-ethinyl estradiol does not cause significant changes to TDF plasmatic levels. This combination is well tolerated and requires no dose adjustment.
**Oral opioid analgesic**		
Methadone and buprenorphine [203]	FTR	Fostemsavir and methadone or buprenorphine coadministration shows a lack of clinically significant drug interactions. Fostemsavir does not cause significant changes to both methadone or buprenorphine plasmatic levels, shown with standardized assessments for opiate withdrawal and overdose scale scores. This combination is well tolerated and can be administered without dose adjustments.
**Special conditions**		
Adults ≥ 60 years [157,204]	EVG/COBI/FTC/TAF	Switching to EVG/COBI/FTC/TAF, in virologically suppressed adults aged 60 years or older, is effective (virological suppression is maintained and there is a moderate increase in CD4 cell counts) and safe (lack of treatment-related serious adverse events and improved safety, both in bone mineral density and renal biomarkers) after 48 weeks.
Adults ≥ 65 years virologically suppressed [157,158,159,160,161,162,163,164,165,166,167,168,169,170,171,172,173,174,175,176,177,178,179,180,181,182,183,184,185,186,187,188,189,190,191,192,193,194,195,196,197,198,199,200,201,202,203,204]	BIC/FTC/TAF	Switching to BIC/FTC/TAF, in virologically suppressed adults aged 65 years or older, is effective (97.7% and 90.7% virologic suppression at weeks 24 and 48, respectively) and safe (not grade 3–4, serious drug-related adverse events or deaths) after 48 weeks.
**Statin**		
Pitavastatin [205,206]	ATV/RTV DRV/RTV, LPV/RTV	Due to pitavastatin being marginally metabolized by the hepatic CYP3A4 enzyme, this statin can be used (4 mg daily) in the setting of a complex background ART and no clinically significant pharmacokinetic interactions have been noted with its coadministration with ATV/RTV, LPV/RTV, or DRV/RTV. Therefore, there are no significant changes in the plasma levels of PIs and pitavastatin; these combinations are safe and no dose adjustments are necessary.
Pitavastatin [205,206]	DOR, EFV, ETR, NVP, RPV	Due to pitavastatin being marginally metabolized by the hepatic cytochrome P450 enzyme, this statin can be used (4 mg daily) in the setting of a complex background ART, and no clinically significant pharmacokinetic interactions have been noted with its coadministration with DOR, EFV, ETR, NVP, or RPV. Therefore, there are no significant changes in the plasma levels of these ARVs and pitavastatin; these combinations are safe and no dose adjustments are necessary.
Pravastatin [205,206]	DOR, NVP, RPV	Due to pravastatin being marginally metabolized by the hepatic cytochrome P450 enzyme, this statin can be used (40 mg daily) in the setting of a complex background ART, and no clinically significant pharmacokinetic interactions have been noted with its coadministration with DOR, NVP, or RPV. Therefore, there are no significant changes in the plasma levels of these ARVs and pravastatin; these combinations are safe and no dose adjustments are necessary.

ABC: abacavir; ABC/AZT: abacavir/zidovudine; ABC/3TC: abacavir/lamivudine; ARV: antiretroviral; ATV/RTV: atazanavir/ritonavir; AZT: zidovudine; BIC: bictegravir; BIC/TAF/FTC: bictegravir/tenofovir alafenamide/emtricitabine; CAB: cabotegravir; DOR: doravirine; DRV: darunavir; DRV/RTV: darunavir/ritonavir; DTG: dolutegravir; DTG/ABC/3TC: dolutegravir/abacavir/lamivudine; EFV: efavirenz; ETR: etravirine; EVG: elvitegravir; EVG/COBI: elvitegravir/cobicistat; GAHT: gender-affirmation hormone therapy; HIV: human immunodeficiency virus; InSTIs integrase strand transfer inhibitors; LPV/RTV: lopinavir/ritonavir; NNRTIs: non-nucleoside reverse transcriptase inhibitors; NVP: nevirapine; PIs: protease inhibitors; RAL: raltegravir; RPV: rilpivirine; TAF: tenofovir alafenamide; TDF: tenofovir disoproxil-fumarate; TDF/FTC: tenofovir disoproxil-fumarate/emtricitabine; TDF/FTC/RPV: tenofovir disoproxil-fumarate/emtricitabine/rilpivirine; 3TC: lamivudine.

## Data Availability

Data are contained within this article.
